# High Parathyroid Hormone Rather than Low Vitamin D Is Associated with Reduced Event-Free Survival in Childhood Cancer

**DOI:** 10.1158/1055-9965.EPI-24-0477

**Published:** 2024-08-14

**Authors:** Corinna Grasemann, Jakob Höppner, Wolfgang Högler, Stephan Tippelt, Maximilian Grasemann, Desiree Grabow, Gunnar Cario, Martin Zimmermann, Martin Schrappe, Dirk Reinhardt, Michael M. Schündeln

**Affiliations:** 1 Division for Rare Diseases, Department of Pediatrics, Katholisches Klinikum Bochum, Ruhr-University Bochum, Bochum, Germany.; 2 Endocrine Unit, Department of Medicine, Massachusetts General Hospital and Harvard Medical School, Boston, Massachusetts.; 3 Department of Paediatrics and Adolescent Medicine, Johannes Kepler University Linz, Linz, Austria.; 4 Division of Pediatric Hematology and Oncology, Department of Pediatrics III, University Hospital Essen, University of Duisburg-Essen, Essen, Germany.; 5 German Childhood Cancer Registry, Division of Childhood Cancer Epidemiology, Institute of Medical Biostatistics, Epidemiology and Informatics (IMBEI), University Medical Center of the Johannes Gutenberg University Mainz, Mainz, Germany.; 6 Pediatric Hematology/Oncology, University Hospital Schleswig-Holstein, Campus Kiel, Kiel, Germany.; 7 Department of Pediatric Hematology and Oncology, Hannover Medical School, Hannover, Germany.

## Abstract

**Background:**

Vitamin D deficiency is linked to poor cancer outcomes but the impact of its consequence, elevated parathyroid hormone (PTH), remains understudied. PTH receptor activation influences cancer progression *in vitro*, yet the effect of elevated PTH on pediatric cancer survival is unexamined.

**Methods:**

This retrospective study examines associations between PTH, 25-OH vitamin D (25OHD), and event-free survival (EFS) and overall survival (OS) in patients with pediatric cancer. Laboratory data from 4,349 patients (0–18 years) at a tertiary pediatric cancer unit were analyzed for the highest PTH and lowest 25OHD levels at diagnosis and the following 5 years. Data on relapse, secondary malignancies, and mortality were stratified by PTH levels above/below the cohort median (47 pg/mL) and 25OHD levels ≤30 nmol/L. EFS and OS were analyzed and hazard ratios (HR) were calculated for the entire cohort and six cancer subgroups.

**Results:**

PTH and 25OHD values were available for 1,286 patients (731 male). Higher PTH associated with inferior EFS in primary malignant brain tumors [HR, 1.80 (1.19–2.72)], embryonal malignancies [HR, 2.20 (1.1–4.43)], and lymphatic malignancies [HR 1.98 (1.05–3.72)]. Vitamin D deficiency associated with inferior EFS in embryonal malignancies [HR 2.41 (1.24–4.68)]. In a multivariate Cox model, only higher PTH remained significant for inferior EFS.

**Conclusions:**

Elevated PTH may indicate adverse outcomes in certain pediatric cancers.

**Impact:**

This study identifies elevated parathyroid hormone as a potential marker for poor outcomes in patients with pediatric cancer, emphasizing the need for adequate vitamin D and calcium management.

## Introduction

Over the past two decades observational, preclinical, and clinical studies suggested that vitamin D deficiency may increase the risk for the development of different malignancies while sufficient vitamin D levels may have antitumoral effects (1–4). Proposed mechanisms underlying these anticancer effects include antiproliferative and anti-inflammatory action, induction of apoptosis, stimulation of differentiation, inhibition of invasion and metastasis, and inhibition of angiogenesis, presumably through direct action on the vitamin D receptors (1, 5).

In humans, vitamin D status is mainly determined by exposure of the unprotected skin to sunlight, by (oral) supplementation and, to a small degree, by diet. Vitamin D insufficiency in children and adolescents, commonly defined as 25-OH vitamin D (25OHD) serum levels below 50 nmol/L or deficiency, less clearly defined as 25OHD ranging from 30 nmol/L (6) to 37.5 nmol/L (7) is common especially during winter and spring in the northern hemisphere (2, 8–10).

Despite the common occurrence of vitamin D deficiency, a large body of experimental and observational evidence suggests it poses a risk factor for cancer development, relapse, and inferior outcome especially in adults (1, 11, 12).

However, from an endocrine perspective, active vitamin D (calcitriol) is a steroid hormone which primarily increases intestinal calcium absorption and thus controls the maintenance of a sufficient body calcium pool. Most importantly, this calcium pool is tightly controlled: declining serum calcium levels result in the release of parathyroid hormone (PTH). Via its end-organ receptor PTH1R (13), PTH indirectly stimulates osteoclasts to ensure calcium retrieval from the skeleton through increased bone resorption, in addition to maximizing intestinal calcium absorption by stimulating the hydroxylation of 25-OHD into calcitriol [1,25(OH)2D; refs. 2, 14]. Therefore, in vitamin D deficiency and in dietary calcium deficiency, PTH elevation and the development of secondary hyperparathyroidism is observed frequently (15, 16).

The two PTH receptors PTH1R and PTH2R are expressed in many tissues including different bone, kidney, and parathyroid cells as well as gut, adrenal, and mesenchymal stem cells, T cells, and in the brain (13).

Historically, a connection between PTH and cancer has been described when PTH-related peptide (PTHrP) was identified in 1988 by three groups as the hormone responsible for humoral hypercalcemia in cancer (17). PTHrP is encoded by *PTHLH* and exhibits paracrine and endocrine actions via the PTH1R receptor. The hormone was studied extensively in the years following its discovery and was found to be relevant in many different types of cancer (18, 19). Importantly, the role of PTHrP in cancer is not limited to the skeletal effects which result in the development of hypercalcemia but it was found to modulate initiation, progression, and metastasis, e.g., in breast cancer (20), in colon cancer (21) and in neuroblastoma tumors *in vitro* (22).

In addition to PTHrP, a potential carcinogenic effect of PTH itself presented when the phase 3 trials for teriparatide (PTH1-34) were prematurely terminated because Fischer rats in the preclinical setting had developed osteosarcoma in a dose-dependent manner when treated with supratherapeutic doses of teriparatide (23). While therapeutic doses did not lead to the development of osteosarcomas, a boxed warning about this risk was in place for almost 20 years for the use of teriparatide (24).

Over the last decades, research focused on cancer risk from vitamin D deficiency, with a presumed mechanism of action via reduced vitamin D receptor stimulation. However, an alternative hypothesis, the stimulation of PTH receptors through secondary hyperparathyroidism has not been sufficiently explored to date. Despite the knowledge that low vitamin D causes secondary hyperparathyroidism and that PTH exerts direct stimulatory actions on many cells and tissues, to the best of our knowledge, PTH has not been directly studied in the context of outcome in childhood malignancies. Hence, the aim of the present study was to investigate a possible association of PTH levels on cancer outcome in a large pediatric cohort.

## Materials and Methods

Laboratory data of 4,349 patients with any childhood malignancy or with a primary benign brain tumor, who were managed between January 2000 and July 2021 at a tertiary pediatric cancer unit at the Department of Paediatrics at the University Hospital Essen were obtained. Data were screened for available PTH and 25OHD levels in each patient for a 5-year period starting from the time of initial diagnosis. Measurement of PTH and 25OHD was integrated into the routine follow-up of patients with pediatric cancer starting in 2012. Subsequently, these measurements were conducted annually as part of a study investigating endocrine and osteological late effects (Acronym: BONE-OK; DRKS#: 00009841)

In each case, the highest plasma PTH and the lowest serum 25OHD during the 5-year period were identified and used for further analyses.

To investigate effects of higher versus lower PTH levels in this cohort, the median PTH of the entire cohort was calculated and used as a cutoff to assign cases to either the group with PTH levels at or above the cohort median (PTH+ group) or the group with PTH levels below the cohort median (PTH− group).

For the analysis on vitamin D, the data were grouped according to the presence (25OHD− group) or absence (25OHD+ group) of vitamin D deficiency (serum 25OHD ≤ 30 nmol/L).

From chart review, the following information were retrieved: sex, date of birth (DOB), diagnosis (malignancy), date of first diagnosis, age at diagnosis, event-free (EFS) and overall (OS) survival, date of first relapse, secondary malignancy, and death. These data were crosschecked and complemented by registry data from the German Childhood Cancer Registry, a nationwide population-based registry on childhood and adolescent cancer (Deutsches Kinderkrebsregister; ref. 25) from the Akute lymphatische Leukämie – Berlin-Frankfurt-Münster study center.

Patients were assigned to the following six diagnostic subgroups according to the biological origin of their initial diagnosis: lymphatic malignancy, malignant primary brain tumor, benign primary brain tumor, embryonal malignancy, sarcoma, myeloid malignancy, and the group “other.” The group “other” comprises smaller groups of patients with different underlying malignancies and was not considered for further analysis due to its heterogeneity.

The study was conducted according to the Helsinki principles and was approved by the Research Ethics Committee of the Medical Faculty of the University Duisburg-Essen (# 23-11270-BO).

### Statistical analyses

Statistical analyses and data visualization were performed in R (version 4.3.0; packages survival version 3.5–5, survminer 0.4.9, SubgrPlots 0.1.3, Table 1 1.4.34, ggplot2, knitr2, https://CRAN.R-project.org/package=survminer. Published online 2021:2021; refs. 26–28).

**Table 1.** Clinical characteristics of the group of patients with PTH levels below the group-median (47 pg/mL; PTH− group, first column), the patients with PTH levels ≥ the median (PTH+ group, middle column), and the entire group (last column).

	PTH < Median (PTH−; *N* = 634)	PTH ≥ median (PTH+; *N* = 634)	P value	Entire cohort (*N* = 1,268)
Number of PTH measurements				
Mean (SD)	2.18 (2.02)	4.16 (3.16)	<0.001	3.17 (2.83)
Median (min, max)	1.00 (1.00, 14.0)	4.00 (1.00, 22.0)		2.00 (1.00, 22.0)
Age at diagnosis (years)				
Mean (SD)	7.34 (5.54)	8.33 (5.30)	0.0011	7.84 (5.44)
Median (min, max)	5.94 (0, 18.3)	8.04 (0.0438, 17.9)		7.04 (0, 18.3)
Follow-up time (years)				
Mean (SD)	5.14 (3.92)	5.67 (3.57)	0.012	5.41 (3.76)
Median (min, max)	4.51 (0.05, 17.30)	5.27 (0.09, 18.18)		4.93 (0.05, 18.18)
Sex				
Male	376 (59.3%)	355 (56.0%)	0.256	731 (57.6%)
Female	258 (40.7%)	279 (44.0%)		537 (42.4%)
Diagnostic groups				
Lymphatic L/L	110 (17.4%)	194 (30.6%)	<0.001	304 (24.0%)
Malignant PBT	151 (23.8%)	107 (16.9%)		258 (20.3%)
Benign PBT	133 (21.0%)	105 (16.6%)		238 (18.8%)
Embryonal	99 (15.6%)	78 (12.3%)		177 (14.0%)
Sarcoma	93 (14.7%)	75 (11.8%)		168 (13.2%)
Myeloid	28 (4.4%)	54 (8.5%)		82 (6.5%)
Other	20 (3.2%)	21 (3.3%)		41 (3.2%)
Highest PTH (pg/mL)				
Mean (SD)	32.6 (9.01)	81.6 (55.8)	<0.001	57.1 (46.9)
Median (min, max)	33.0 (6.40, 46.9)	68.5 (47.0, 1,030)		47.0 (6.40, 1,030)
Corresponding 25OHD (nmol/L)				
Mean (SD)	47.6 (23.9)	41.3 (25.7)	<0.001	44.5 (25.0)
Median (min, max)	44.8 (10.0, 146)	36.3 (10.0, 248)		41.0 (10.0, 248)
Time from diagnosis to highest PTH (years)				
Mean (SD)	1.60 (1.52)	1.75 (1.39)	0.78	1.67 (1.46)
Median (min, max)	1.01 (0.00, 5.00)	1.37 (0.00, 4.99)		1.25 (0.00, 5.00)
Time from highest PTH to event (years)				
Mean (SD)	−0.32 (1.87)	−0.13 (2.14)	0.374	−0.22 (2.02)
Median (min, max)	−0.27 (−4.68, 7.32)	−0.22 (−4.81, 9.69)		−0.26 (−4.81, 9.69)
Lowest 25OHD (nmol/L)				
Mean (SD)	41.1 (22.7)	29.3 (20.2)	<0.001	35.2 (22.3)
Median (min, max)	35.8 (9.25, 146)	24.5 (8.25, 230)		29.5 (8.25, 230)
Time from diagnosis to lowest 25OHD (years)				
Mean (SD)	2.01 (2.54)	2.40 (2.58)	0.008	2.20 (2.56)
Median (min, max)	0.75 (0.00, 12.72)	1.30 (0.00, 14.93)		1.01 (0.00, 14.93)
5-year OS				
Percent	88.7	87.9	0.284	88.3
% CI	85.9–91.6	85.1–90.7		86.3–90.3

NOTE: PTH measurements were obtained at any time during the 5 years following the initial diagnosis. The number of PTH measurements during follow up, age, sex and diagnostic groups, the highest PTH, the corresponding 25OHD serum level, the lowest 25OHD, and the distribution as well as *P* values for the PTH+ and PTH− group are provided. The timespan between the intial diagnosis and the measurement of the highest PTH/lowest 25OHD and the time between the highest PTH and the event are also provided.

Abbreviations: PTH, parathyroid hormone; 25OHD, 25-hydroxy vitamin D; L/L, leukemia or lymphoma; PBT, primary brain tumor.

Values are expressed as the mean ± standard deviation (SD), median, and range unless stated otherwise.

EFS was defined as the time from initial diagnosis to the occurrence of first relapse, secondary malignancy, or death, whichever occurred first. OS was defined as time from initial diagnosis to death. Of note, nonresponse to treatment was not counted as an event in the analysis. The Kaplan–Meier method was used to estimate overall survival (29); differences between groups were compared using the two-sided log-rank test (Mantel–Cox; ref. 30). To visualize the effect of PTH as a continuous variable on the time-to-event outcome, the R package contsurvplot was applied (31). The Cox proportional hazard model was used for univariate and multivariate analyses (32). The timespan from first diagnosis of malignancy to death/event or last contact in case of no event was used as time variable. Sex, age at diagnosis, the presence of vitamin D deficiency, and presence of a plasma PTH concentration below or above the median during the observation period were used as covariables for multivariate analyses. Forest plots for disease-subgroup analyses were modified according to Cuzick (28) to display the difference in the effect of higher PTH and vitamin D deficiency on EFS/OS in subgroups in contrast to the average overall effect in the whole cohort. Differences in the distribution of individual parameters among patient subsets were analyzed using the *χ*^2^ test for categorized variables and the Mann–Whitney *U* test for continuous variables. For all tests, statistical significance was presumed at *P* < 0.05.

### Data availability

The data generated in this study are available upon request from the corresponding author.

## Results

### Cohort characteristics

In total in 1,268 cases (731 male) with suitable measurements of plasma PTH (median number of measurements per case: 2, range 1–22) were identified. Out of those, at least one 25OHD measurement was also available in 1,252 patients in the 5-year period of interest.

The median PTH of the entire group was identified as 47 pg/mL. A total of 634 patients were assigned to the group with PTH levels at or above the median (PTH+ group) and 634 had PTH readings below the median (PTH− group). In Table 1, clinical characteristics and highest PTH and lowest 25OHD levels (mean, median, min, max) of the group are provided (for details see Table 1).

The PTH+ group was older (8.33 + 5.3 years) than the PTH– group (7.34 + 5.54 years, *P =* 0.001), and had more PTH readings (median = 2) than the PTH− group (median = 1). As expected, 25OHD levels were significantly lower in the PTH+ group compared to the PTH− group (for details see Table 1).

For the analyses of 25OHD, data from 1,252 patients (of the 1,268) were available. In 638 patients, the lowest 25OHD concentration was <30 nmol/L (25OHD− group) and in 614 the lowest concentration was >30 nmol (25OHD+ group).

Patients were assigned to the six diagnostic cancer groups as following: lymphatic malignancy (*n* = 304), malignant primary brain tumor (*n* = 258), benign primary brain tumor (*n* = 238), embryonal malignancy (*n* = 177), sarcoma (*n* = 168), myeloid malignancy (*n* = 82), and the group “other” (*n* = 41). A detailed list of all diagnoses and the assignment to the corresponding groups is available in Supplementary Table S1.

### Overall EFS and OS in the entire cohort

As expected, the 5-year EFS/OS differed depending on the underlying malignancy. For detailed information on the 5-year EFS/OS refer to Table 2 and Supplementary Fig. S1A and S1B. The highest 5-year EFS was seen in the lymphatic malignancies [82.1, 77.6%–86.8% (95% CI)] while the lowest 5-year EFS (56.2, 49.7%–64.2%) and OS (73.2, 66.9%–80.2%) was seen in malignant PBT.

**Table 2.** Five-year OS and EFS for the group of patients with PTH levels below the median (47 pg/mL) of the group (PTH− group, first column), the patients with PTH levels ≥ the median (PTH+ group, second column), the entire PTH-group (third column), the group with deficient (25OHD < 30 nmol/L) vitamin D levels (25OHD− group, fourth column), the group with sufficient vitamin D levels (25OHD+ group, last column).

	PTH < Median (PTH−)	PTH ≥ Median (PTH+)	Entire Group(= PTH+ and PTH−)	25OHD < 30 (25OHD−)	25OHD > 30 (25OHD+)
Group size	*N* = 634	*N* = 634	*N* = 1,268	*N* = 638	*N* = 614
Deaths	*N* = 58	*N* = 78	*N* = 136	*N* = 54	*N* = 78
5-year OS (%)	88.7	87.9	88.3	88.1	88.8
Percent/95% CI	85.9–91.6	85.1–90.7	86.3–90.3	85.4–90.9	85.9–91.9
Events	*N* = 159	*N* = 188	*N* = 347	*N* = 160	*N* = 179
5-year EFS (%)	71.0	68.7	69.7	70.9	68.7
Percent/95% CI	67.1–75.0	64.9–72.7	67.0–72.6	67.3–74.8	64.7–73.1
Benign PBT	*N* = 133	*N* = 105	*N* = 238	*N* = 126	*N* = 111
Deaths	*N* = 4	*N* = 3	*N* = 7	*N* = 4	*N* = 3
5-year OS (%)	97.8	97.5	97.6	99.1	95.7
Percent/95% CI	94.7–100	94.1–100	95.3–100	97.4–1	91.1–100
Events	*N* = 53	*N* = 33	*N* = 86	*N* = 45	*N* = 40
5-year EFS (%)	57.4	67.7	62.3	68.2	55.3
Percent/95% CI	49.4–67.4	59.0–77.7	56.1–69.1	60.4–77.0	46.0–66.6
Embryonal	*N* = 99	*N* = 78	*N* = 177	*N* = 53	*N* = 120
Deaths	*N* = 4	*N* = 6	*N* = 10	*N* = 3	*N* = 7
5-year OS (%)	96.3	93.9	95.0	91.0	96.8
Percent/95% CI	92.3–100	88.1–100	91.3–98.8	82.8–100	93.4–100
Events	*N* = 12	*N* = 23	*N* = 35	*N* = 17	*N* = 18
5-year EFS (%)	83.3	66.8	75.1	60.3	81.7
Percent/95% CI	74.6–93.0	56.4–79.2	68.0–82.9	47.2–76.9	73.9–90.3
Lymphatic L/L	*N* = 110	*N* = 194	*N* = 304	*N* = 195	*N* = 104
Deaths	*N* = 4	*N* = 12	*N* = 16	*N* = 5	*N* = 10
5-year OS (%)	95.5	93.1	94.0	94.2	94.4
Percent/95% CI	91.3–100	89.3–97.0	91.1–96.9	90.7–97.8	89.6–99.4
Events	*N* = 13	*N* = 40	*N* = 53	*N* = 15	*N* = 35
5-year EFS (%)	87.7	78.9	82.1	82.0	84.5
Percent/95% CI	81.4–94.5	73.0–85.3	77.6–86.8	76.5–87.9	77.2–92.4
Malignant PBT	*N* = 151	*N* = 107	*N* = 258	*N* = 107	*N* = 149
Deaths	*N* = 24	*N* = 32	*N* = 56	*N* = 21	*N* = 33
5-year OS (%)	74.8	71.0	73.2	68.4	78.9
Percent/95% CI	66.2–84.5	61.6–81.7	66.9–80.2	59.2–78.9	70.4–88.5
Events	*N* = 40	*N* = 51	*N* = 91	*N* = 46	*N* = 43
5-year EFS (%)	64.3	46.9	56.2	58.3	55.1
Percent/95% CI	55.4 -74.5	37.3–58.9	49.7–64.2	49.0–69.4	45.6–66.6
Myeloid malignancy	*N* = 28	*N* = 54	*N* = 82	*N* = 58	*N* = 23
Deaths	*N* = 4	*N* = 8	*N* = 12	*N* = 3	*N* = 9
5-year OS (%)	85.3	86.3	85.9	85.1	87.0
Percent/95% CI	73.0–99.7	77.3–96.3	78.6–94.0	76.1–95.2	74.2–100
Events	*N* = 8	*N* = 14	*N* = 22	*N* = 7	*N* = 14
5-year EFS (%)	70.6	74.6	73.2	74.1	69.6
Percent/95% CI	55.3–90.0	63.5–87-6	64.0–83.7	63.3–86.8	53.1–91.2
Sarcoma	*N* = 93	*N* = 75	*N* = 168	*N* = 77	*N* = 88
Deaths	*N* = 17	*N* = 12	N = 29	*N* = 15	*N* = 13
5-year OS (%)	76.4	76.6	76.5	78.5	75.3
Percent/95% CI	67.1–86.9	65-0.7–89.4	69.2–84.6	68.6–89.8	65.0–87.3
Events	*N* = 30	*N* = 22	*N* = 52	*N* = 26	*N* = 24
5-year EFS (%)	61.9	64.9	63.1	64.4	62.0
Percent/95% CI	51.6–74.1	54 0.0–78.1	55.4–71.9	53.7–77.1	51.2–75.0
Other	*N* = 20	*N* = 21	*N* = 41	*N* = 22	*N* = 19
Deaths	*N* = 1	*N* = 5	*N* = 6	*N* = 3	*N* = 3
5-year OS (%)	94.1	78.5	85.9	89.6	82.2
Percent/95% CI	83.6–100	61.8–99.8	75.0–98.2	77.0–100	65.8–100
Events	*N* = 3	*N* = 5	*N* = 8	*N* = 4	*N* = 4
5-year EFS (%)	81.4	80.4	80.7	83.2	77.8
Percent/95% CI	64.5–100	64.9–99.6	68.7–94.7	67.4–100	60.8–99.6

NOTE: PTH and 25OHD measurements were obtained at any time during the 5-years following the initial diagnosis.

Abbreviations: L/L, leukemia or lymphoma; PBT, primary brain tumor.

### 25OHD and PTH and EFS/OS

**Figure 1.**
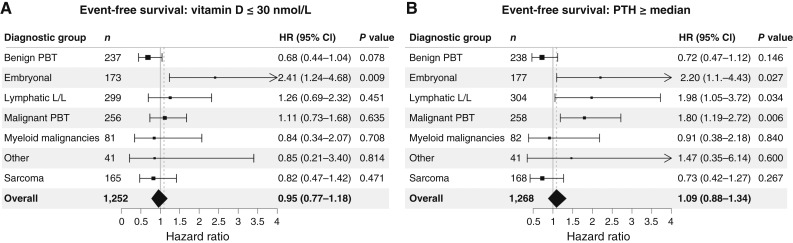
**A** and **B,** EFS in the entire group and in six diagnostic groups. HR and 95% confidence interval (CI) for the presence of a vitamin D deficiency (25 OH vitamin D level at or below 30 nmol/L) at any time during the 5 years following the initial diagnosis is shown in **A**, and for the presence of PTH levels at or above 47 pg/mL (= median of the group) is shown in **B**. A univariate analysis was performed.

Only in the group of embryonal malignancies was the mean EFS inferior in the 25OHD− group (25OHD < 30 nmol/L) compared to the 25OHD+ group [*N* = 173, 5-year EFS: 60.3, 47.2%–76.9% (95% CI) vs. 81.7, 73.9%–90.3%] with an increased hazard ratio (HR) of 2.41 (1.24–4.68) Fig. 1A and Table 2. Except for the aforementioned group of embryonal malignancies, vitamin D deficiency during follow-up was not associated with an inferior EFS or OS in the entire cohort or any of the other subgroups in a univariate Cox regression model.

PTH levels ≥47 pg/mL (PTH+ group) at any time within the first 5 years following the cancer diagnosis were associated with significantly inferior EFS in the groups: embryonal malignancies, lymphatic malignancies, and malignant primary brain tumors (see below and Fig. 1B). OS was not inferior in any group.

In the group of embryonal malignancies (*N* = 177), the mean 5-year EFS was lower in PTH+ (66.8%, 57.9%–56.4%) compared to the PTH− group (83.3%, 74.6%–93.0%). Correspondingly, the hazard ratio was increased in the univariate Cox proportional hazard model in the presence of higher PTH [EFS HR 2.20 (1.1–4.43)].

In the group of lymphatic malignancies (*N* = 304), the mean 5-year EFS was 78.9% (73.0%–85.3%) in PTH+ compared to 87.7% (81.4.–94.5%) in the PTH− group. The hazard ratio was increased [(HR) 1.98 (1.05–3.72)].

In the group of malignant primary brain tumors (*N* = 258) the mean 5-year EFS was 46.9% (37.3%–60.0%) in PTH+ compared to the PTH− group (64.3%; 55.4.–74.5%). The corresponding hazard ratio was increased [(HR) 1.80 (1.19–2.72)].

**Figure 2.**
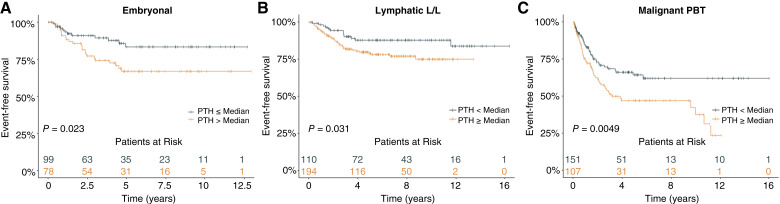
**A–C,** Kaplan–Meier plots showing EFS in subgroups. The Kaplan–Meier plots show the EFS in the groups of embryonal malignancies (**A**) lymphatic malignancies (**B**) and malignant primary brain tumor (PBT; **C**) for patients with PTH at/above the median (47 pg/mL) of the entire group (orange line) and patients with PTH levels below the median (gray line). For the analyses, the highest PTH at diagnosis or during the following 5-year period was used. *P* values from paired log rank tests are provided.

**Figure 3.**
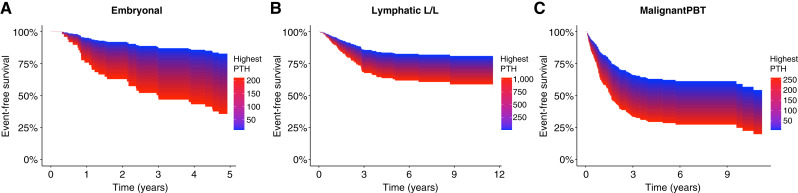
**A–C,** EFS in subgroups with PTH as a continuous variable. Visualizing of the highest measured PTH on time-to-event (as EFS) in a model assuming a linear PTH-effect in the subgroups of embryonal malignancies (**A**) lymphatic malignancies (**B**) and malignant primary brain tumor (PBT; **C**). For the analyses, the highest PTH at diagnosis or during the following 5-year period was used.

The three corresponding Kaplan–Meier plots for EFS are displayed in Fig. 2A–C. Kaplan–Meier plots for OS and EFS for the entire cohort and all subgroups are displayed as Supplementary Figs. S2A–S2H and S3A–S3H. In Fig. 3A–C survival for the three groups with PTH as a continuous variable in a model assuming a linear PTH-effect are presented.

The HR for OS was not increased in any group.

**Figure 4.**
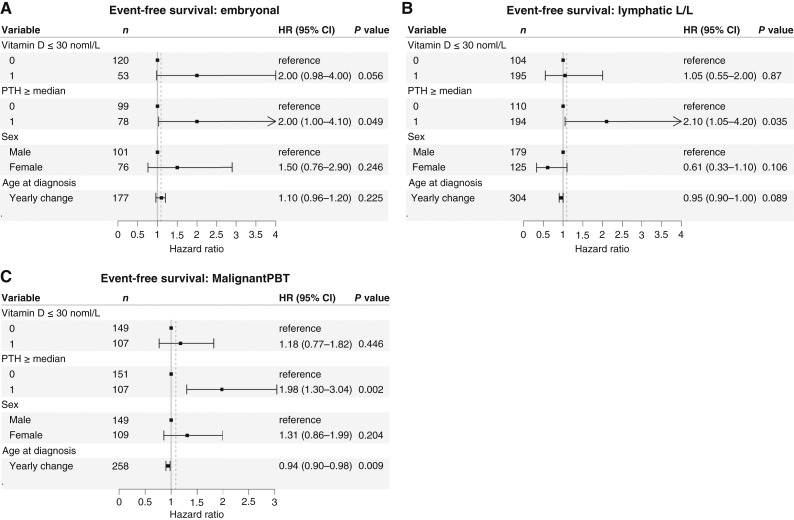
**A–C,** Multivariate Cox proportional hazard model for EFS. The variables “vitamin D deficiency” (defined by a 25 OH vitamin D level < 30 nmol/L), “higher PTH” (defined by a PTH level > 47 pg/mL), sex and age at diagnosis were investigated for EFS in the groups: embryonal malignancies (**A**) lymphatic malignancies (**B**) and malignant primary brain tumor (PBT; **C**).

A multivariate analysis of EFS and OS (dependent variable) including the covariables age at diagnosis, sex, presence/non-presence of PTH ≥47 pg/mL and of vitamin D deficiency showed a significant association with inferior EFS in the disease groups: embryonal malignancies, lymphatic malignancies, and malignant PBT for elevated PTH only Fig. 4A–C.

## Discussion

This study is the first to investigate an effect of higher PTH on EFS/OS in childhood malignancies, independent of vitamin D. While it is often postulated that vitamin D deficiency increases the risk of cancer development and for inferior outcome (1), studies on effects of PTH are sparse despite its stimulatory effect via PTH1R not only on excessive bone resorption but also other cell types (via PTH1R and PTH2R).

Here, we provide evidence linking higher PTH levels at diagnosis or within the subsequent 5-year period to inferior EFS, but unchanged OS, in childhood embryonal and lymphatic malignancies, as well as in childhood malignant primary brain tumors. These findings suggest an increased risk of relapse and second malignancies in patients with pediatric cancer with elevated PTH levels within this large pediatric cancer cohort.

Hyperparathyroidism is a common finding in the winter and spring in northern latitudes and even more so in survivors of childhood cancer, as reported by many groups (9, 33–37). Of note, the actual cutoff to define hyperparathyroidism is heavily debated, in particular in children (38, 39). PTH secretion rises from its baseline to counter decreasing calcium levels in the blood. As calcium availability depends on the vitamin D status as well as the nutritional intake of calcium, it has been argued that normative values for PTH should be established in calcium- and vitamin D–sufficient cohorts only. Indeed, studies have shown that the upper limit of normal for PTH may be 20% (40) or even 25% to 35% (41) lower than the commonly used cut-off in current use, if investigated in vitamin D–sufficient subjects (42, 43). Recent retrospective cross-sectional studies (44, 45) also demonstrate an age dependence of serum PTH levels, with lowest values in the youngest age groups. The PTH-25OHD relationship in children differs substantially from adults, in that PTH levels are much lower for the same 25OHD concentrations. (44, 45) Studies specifically reporting pediatric PTH reference data for children are discrepant, likely explained by the differing vitamin D status, dietary intakes, and fasting duration of the study populations. Reported upper limits of normal range from 35 pg/mL (46) to 60 to 80 pg/mL (47). In the absence of an established upper limit for PTH in the age group of interest, we therefore decided to use the median PTH (47 pg/mL) of the entire group as a cutoff for this study. As discussed, this is lower than commonly used threshold for adults of 65 pg/mL.

Data on the association between PTH and the outcome of malignancies in humans are limited, especially in children. In one cross-sectional study on 295 children with a malignant diagnosis, Jackmann and colleagues reported no association of PTH with cancer outcome (16). In adults few studies are available: cancer-related mortality was studied in 1,317 adults (median age 75 years) who participated in the Longitudinal Amsterdam Aging study. In the baseline assessment vitamin D deficiency (25OHD < 50 nmol/L) was present in about 50% and secondary hyperparathyroidism had developed in almost 5%. No association between PTH and survival was reported (48).

However, others have investigated the association of PTH and the outcome in patients with breast cancer (no clear association between PTH and outcome in men, but detrimental effects in mice; refs. 49, 50) and in prostate cancer (51). The latter group reported that baseline PTH levels were negatively associated with OS in patients with prostate cancer in the presence of bone metastases (52). However, when interpreting these results the suppressing effects of hypercalcemia (due to bone metastases) on PTH and the stimulating effects of hypocalcemia due to antiresorptive treatment of bone metastases need to be considered and do likely not allow an evaluation of PTH on survival as an independent marker.

Kang and colleagues studied serum PTH in 115 patients with multiple myeloma (a malignancy arising in the bone marrow from abnormal plasma cells) and found inferior progression-free survival in the group with high PTH levels compared to the group with normal PTH levels. In addition, serum PTH at diagnosis was associated with risk factors and clinical outcome (53).

Inferior EFS was detected in three groups of childhood malignancies that may seem unrelated at first glance. However, in all three groups tumors arise from tissues or cell types with known expression of PTH1R or PTH2R. For the group of lymphatic malignancies it is well established that PTH-signaling is not only involved in the fate of the mesenchymal stem cells of the bone marrow niche (54) but also directly influences T cells (55). In addition, accumulating evidence shows the bone marrow niche is critical to the maintenance and retention of hematopoietic stem cells (HSC), including leukemia stem cells (LSC; refs. 56–60). In fact, LSC-induced microenvironmental reprogramming of the bone marrow niche contributes significantly to leukemogenesis (61). Activation of the PTH receptor on osteoblasts also increases stem cell number (62–64) and pharmacologic use of PTH increases the number of HSCs mobilized into the peripheral blood for stem cell harvests in mice (65, 66). Moreover, Ballen and colleagues (67) showed that PTH was effective in mobilization of HSCs from bone marrow in patients who previously had one or two unsuccessful stem cell–mobilization attempts. Interestingly, we did not observe inferior EFS/OS in the smaller group of children with myeloid malignancies; an observation that warrants further investigation.

Beyond lymphatic malignancies inferior EFS was observed in children with malignant primary brain tumors. As reviewed by Dettori and colleagues (13), there is almost ubiquitous expression of PTH receptors in the central nervous system, opening the possibility for direct actions of PTH on a cellular level.

Furthermore, for the group of to the group of embryonal malignancies it was shown that neuroblastoma cell lines not only respond to PTH in culture (61) but that PTHrP differentially regulates neuroblastoma cell via action on PTH1R (22). Cell lines of retinoblastoma, the entity representing the largest proportion in the group of embryonal malignancies, respond to PTH as well. Phosphorylation of rb is PTH-dependent and E2F3, which is a candidate for tumor progression in retinoblastoma (68) belongs to the group of E2F which is targeted and stimulated by PTH (69).

Together, these experimental data and the observations of our study suggest that higher and elevated PTH levels could alter the fate of tumor cells and the release of leukemic stem cells from the bone marrow, thereby affecting EFS. It is possible that to some degree the previously described deleterious effects of a vitamin D deficiency on cancer outcome may be mediated by elevation of PTH itself.

### Strength and limitations of the study

The present study is by far the largest study of its kind in children, investigating a potentially relevant association. Even in common vitamin D deficiency, it is well known that bone pathology only starts once PTH rises. Similar effects on cancer development may potentially exist that warrant further research on this topic.

The main limitations of this study are caused by the retrospective design: While complete and structured clinical data on EFS and OS were obtained, the laboratory assessments of PTH and 25OHD were not part of routine laboratory evaluation until 2012 and therefore did not follow a protocol but were prompted by clinical standards. Thus, the timing and frequency of the PTH and 25OHD assessments differed between the patients, and PTH was measured more frequently in patients who were found to have developed hyperparathyroidism than in patients without hyperparathyroidism.

Given the hypothesis that elevated PTH levels may impact EFS and OS in patients with cancer, the analysis was focused on the highest PTH level recorded for each patient during the 5-year period from diagnosis. To align with this analysis and investigate the hypothesis that low 25 OHVD is detrimental to EFS/OS, the lowest recorded 25 OHVD was also analyzed.

The reported association of high PTH with lower EFS in some cancers was noted in the absence of a temporal relationship. One possible explanation is that patients diagnosed with elevated PTH at any point during follow-up may have experienced similar unfavorable calcium homeostasis earlier, which went undetected due to the lack of laboratory assessment. Fully addressing this matter would require a prospective study design

In summary, this study highlights the potential role of elevated PTH as a prognostic marker for poor outcomes in patients with pediatric cancer. The findings suggest that higher PTH levels are associated with inferior EFS in certain cancer subtypes, including malignant primary brain tumors, embryonal, and lymphatic malignancies. These results underscore the importance of monitoring PTH levels in patients with pediatric cancer and ensuring adequate vitamin D and calcium intake to potentially mitigate adverse outcomes. This research contributes to the understanding of biochemical markers in cancer prognosis and supports the need for integrated metabolic management in pediatric oncology.

## Supplementary Material

Supplementary Figure 1A -B Event-free and Overall Survival plots for the entire cohort and Subgroups.

Supplemental Figure 2A-H PTH and Overall Survival of the entire group and subgroups.

Supplemental Figure 3 A-H PTH and Event-free Survival of the entire group and subgroups.

Supplemental Table 1 Diagnoses and assignments to subgroups of the entire cohort.
