# Supplementary material for: High Parathyroid Hormone Rather than Low Vitamin D Is Associated with Reduced Event-Free Survival in Childhood Cancer
Source: Cancer Epidemiol Biomarkers Prev. 2024 Aug 14;33(11):1414–22. doi: 10.1158/1055-9965.EPI-24-0477 (PMC11528194; doi:10.1158/1055-9965.EPI-24-0477)
Supplement: Supplementary Figure 1A -B — Event-free and Overall Survival plots for the entire cohort and Subgroups. [file epi-24-0477_supplementary_figure_1a_-b_suppsf1.docx]

**Supplementary Figure 1**


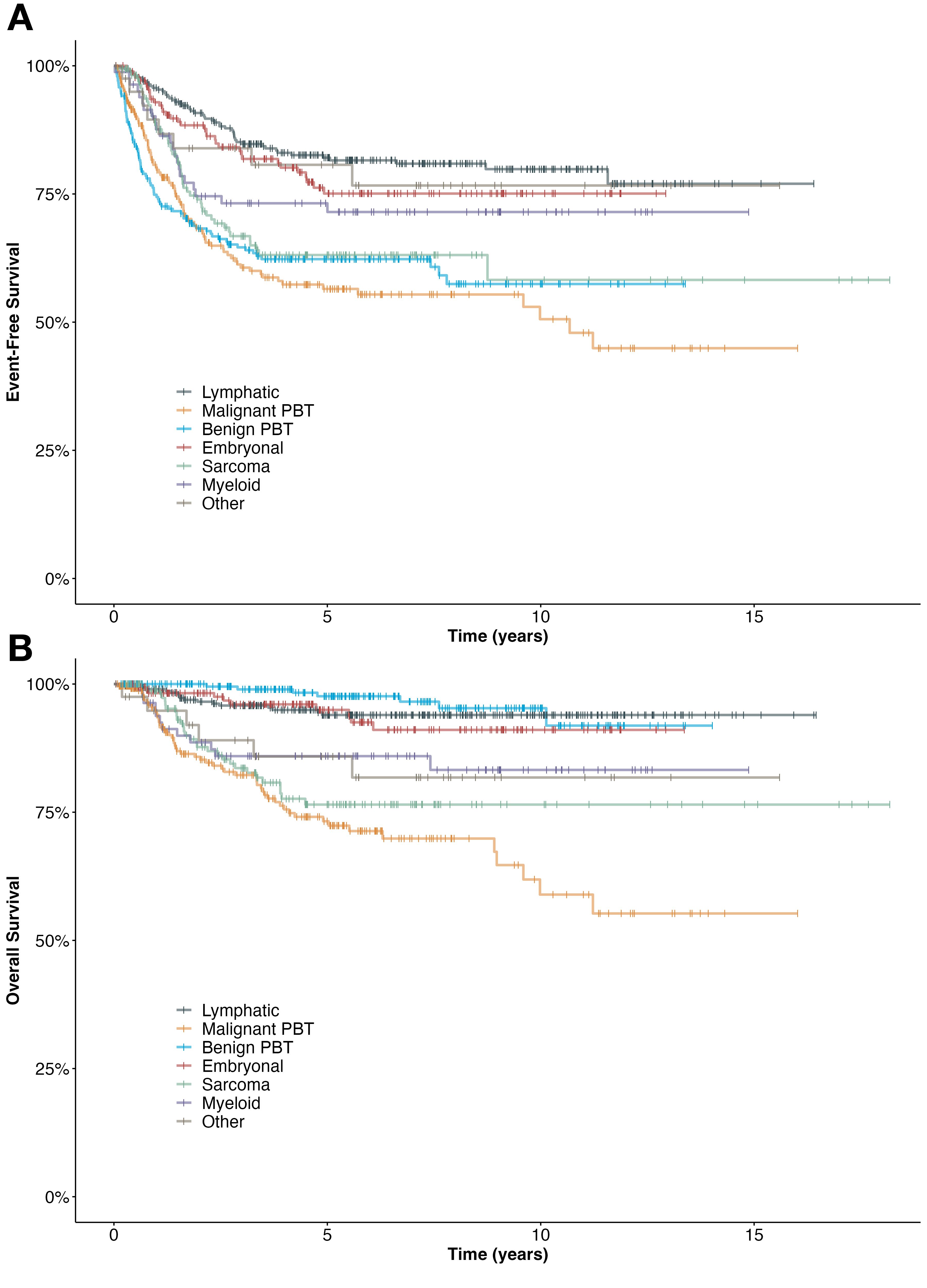


**Supplementary Figure 1 A-B: Event-free and Overall Survival plots of the entire cohort**

Kaplan Meyer curves for event-free Survival (1A) and overall Survival (1B) of the different diagnostic groups of the entire cohort. The diagnostic groups are: lymphatic malignancies (grey), malignant primary brain tumor (PBT) (yellow), benign PBT (blue), embryonal malignancies (red), sarcomas (green), myeloid malignancies (purple) and ‘other’ (brown-grey).
