# Supplementary material for: High Parathyroid Hormone Rather than Low Vitamin D Is Associated with Reduced Event-Free Survival in Childhood Cancer
Source: Cancer Epidemiol Biomarkers Prev. 2024 Aug 14;33(11):1414–22. doi: 10.1158/1055-9965.EPI-24-0477 (PMC11528194; doi:10.1158/1055-9965.EPI-24-0477)
Supplement: Supplemental Figure 3 A-H — PTH and Event-free Survival of the entire group and subgroups. [file epi-24-0477_supplemental_figure_3_a-h_suppsf3.docx]

**Supplementary Figure 3**


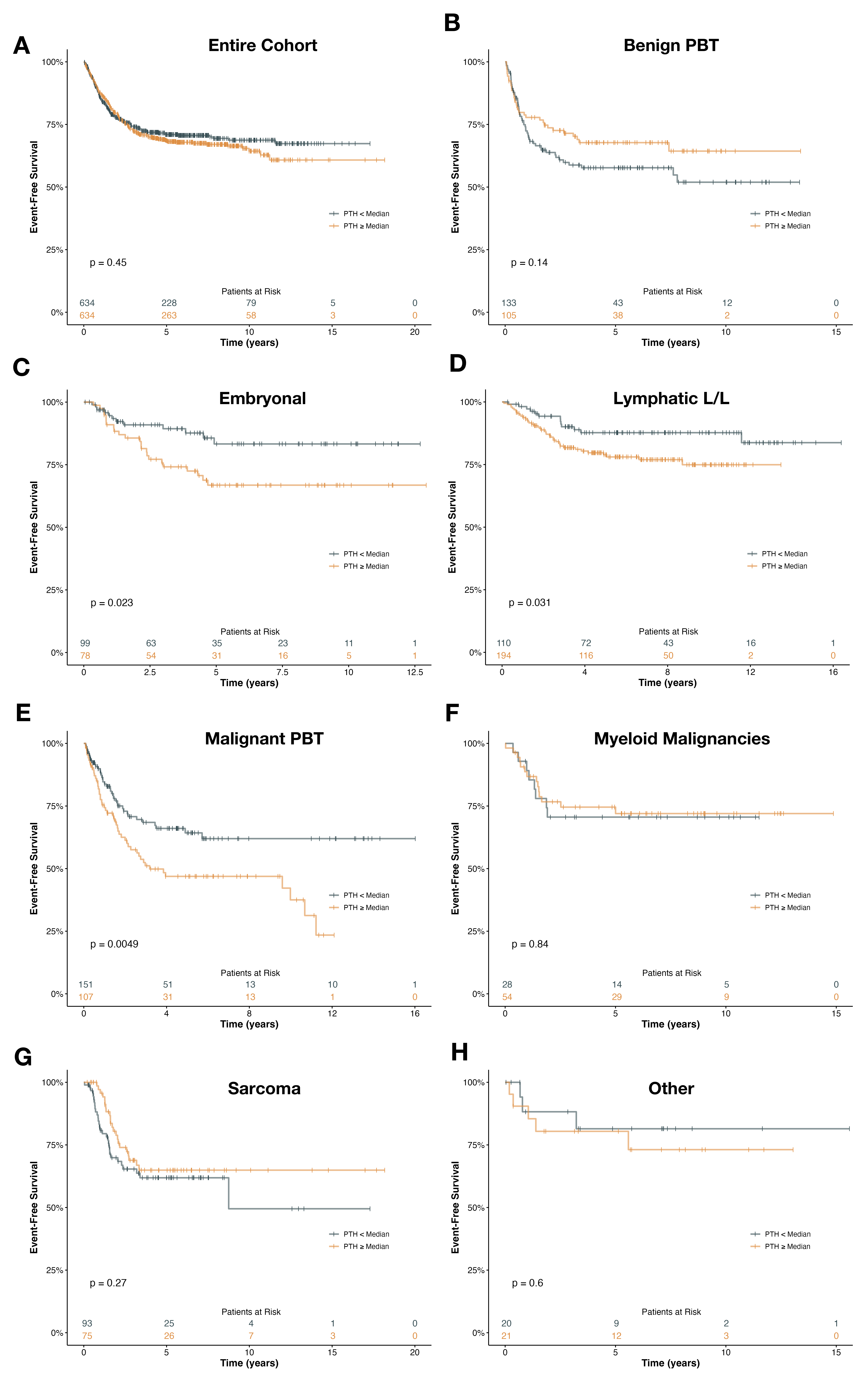


**Supplementary Figure 3**: **Event-free Survival for the entire group and the subgroups depending on the highest measured PTH.** Kaplan Meyer plots showing event free survival in the entire group (3A) and the subgroups: benign primary brain tumor (PBT) (3B), embryonal malignancies (3C) lymphatic malignancies (3D), malignant PBT (3E), Myeloid Malignancies (3F), Sarcomas (3G) and the group ‘Other’ (3H) for patients with PTH > the median (47 pg/ml) of the entire group (orange line) and patients with PTH levels below the median (grey line). For the analyses the highest PTH at diagnosis or during the following 5-year period was used. P values from paired log rank tests are provided.
