# Supplementary material for: High Parathyroid Hormone Rather than Low Vitamin D Is Associated with Reduced Event-Free Survival in Childhood Cancer
Source: Cancer Epidemiol Biomarkers Prev. 2024 Aug 14;33(11):1414–22. doi: 10.1158/1055-9965.EPI-24-0477 (PMC11528194; doi:10.1158/1055-9965.EPI-24-0477)
Supplement: Supplemental Table 1 — Diagnoses and assignments to subgroups of the entire cohort. [file epi-24-0477_supplemental_table_1_suppst1.docx]

**Supplementary Table 1**

| **Disease Group**  Diagnosis | PTH < Median  (PTH-; N=634) | PTH ≥ Median  (PTH+; N=634) | Entire Cohort (N=1268) | Fraction (%) |
| --- | --- | --- | --- | --- |
| **Lymphatic Malignancies** |  |  |  |  |
| ALL | 69 | 107 | 176 | 13,9 |
| ALCL | 5 | 8 | 13 | 1,0 |
| B-NHL | 8 | 20 | 28 | 2,2 |
| Morbus Hodgkin | 26 | 50 | 76 | 6,0 |
| T-NHL | 2 | 9 | 11 | 0,9 |
| **Malignant PBT** |  |  |  |  |
| Astrocytoma WHO° III-IV | 3 | 5 | 8 | 0,6 |
| Ependymoma WHO° III-IV | 38 | 30 | 68 | 5,4 |
| Glioblastoma | 4 | 5 | 9 | 0,7 |
| Other Malignant PBT | 9 | 3 | 12 | 0,9 |
| Medulloblastoma | 54 | 31 | 85 | 6,7 |
| PNET | 2 | 3 | 5 | 0,4 |
| Pons Glioma WHO°III-IV | 6 | 5 | 11 | 0,9 |
| ZNS AT/RT | 19 | 6 | 25 | 2,0 |
| ZNS-Germ Cell Tumor | 15 | 18 | 33 | 2,6 |
| ZNS-PNET | 1 | 1 | 2 | 0,2 |
| **Benign PBT** |  |  |  |  |
| Astrocytoma WHO° I-II | 91 | 87 | 178 | 14,0 |
| Ependymoma WHO° I-II | 15 | 5 | 20 | 1,6 |
| Craniopharyngeoma | 25 | 13 | 38 | 3,0 |
| Meningeoma | 2 | 0 | 2 | 0,2 |
| **Embryonal Tumors** |  |  |  |  |
| Hepatoblastoma | 4 | 8 | 12 | 0,9 |
| Nephroblastoma | 17 | 18 | 35 | 2,8 |
| Neuroblastoma | 14 | 18 | 32 | 2,5 |
| Retinoblastoma | 61 | 31 | 92 | 7,3 |
| Rhabdoid Tumor | 3 | 3 | 6 | 0,5 |
| **Sarcoma** |  |  |  |  |
| Ewingsarcoma | 10 | 20 | 30 | 2,4 |
| Fibrosarcoma | 0 | 2 | 2 | 0,2 |
| Osteosarcoma | 12 | 12 | 24 | 1,9 |
| Rhabdomyosarcoma | 62 | 36 | 98 | 7,7 |
| Soft Tissue Sarcoma | 9 | 5 | 14 | 1,1 |
| **Myeloid** |  |  |  |  |
| AML | 15 | 34 | 49 | 3,9 |
| CML | 3 | 4 | 7 | 0,6 |
| JMML | 1 | 2 | 3 | 0,2 |
| MDS | 9 | 14 | 23 | 1,8 |
| **Others** |  |  |  |  |
| Carcinoma | 6 | 9 | 15 | 1,2 |
| Peripheral Germ Cell Tumor | 5 | 4 | 9 | 0,7 |
| Hepatic Carcinoma | 2 | 1 | 3 | 0,2 |
| Malignant Melanoma | 2 | 2 | 4 | 0,3 |
| Nasopharyngeal Carcinoma | 3 | 3 | 6 | 0,5 |
| Thyroid Carcinoma | 2 | 2 | 4 | 0,3 |
|  |  |  |  |  |

**Supplementary Table 1**. List of all diagnoses and the assignment to the corresponding groups (total number and %) the group of patients with parathyroid hormone (PTH) levels below the group-median (47 pg/ml) (PTH- group, first column), the patients with PTH levels > the median (PTH+ group, middle column) and the entire group (last column). Abbreviations: ALL: acute lymphatic leukemia, ALCL: acute large cell lymphoma, B-NHL: B- non Hodgkin lymphoma, T-NHL: T-non Hodgkin lymphoma, PBT: primary brain tumor, PNET: peripheral neuroectodermal tumor, CNS: Central nervous system, AT/RT: atypical teratoid-rhabdoid tumor, AML: acute myeloid leukemia, CML: chronic myeloid leukemia, JMML: juvenile myelomonocytic leukemia, PBT: primary brain tumor, MDS: myeloid dysplastic syndrome)
